# Supplementary material for: Processing at Phrase Boundaries During Self-Paced Reading
Source: bioRxiv. 2026 Jul 14:2026.07.13.738177. Preprint. [Version 1] doi: 10.64898/2026.07.13.738177 (PMC13404733; doi:10.64898/2026.07.13.738177)
Supplement: Supplement 1 [file NIHPP2026.07.13.738177v1-supplement-1.pdf]

bioRxiv preprint doi: https://doi.org/10.64898/2026.07.13.738177; this version posted July 14, 2026. The copyright holder for this preprint (which was not certified by peer review) is the author/funder, who has granted bioRxiv a license to display the preprint in perpetuity. It is made available under a CC-BY 4.0 International license.

Supplementary figures

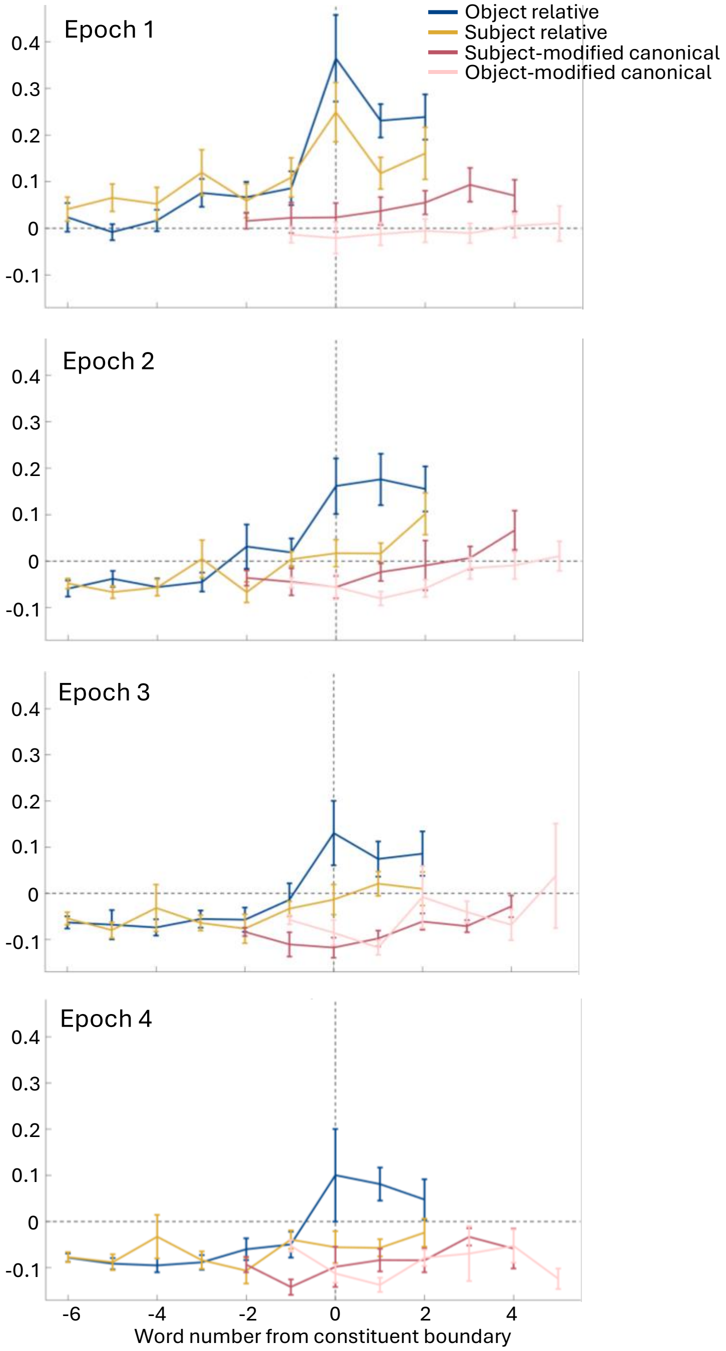

Figure S1. The graphs compare plots per-word response times across the word number from the constituent boundary to describe behavior over four succeeding epoch times.

bioRxiv preprint doi: https://doi.org/10.64898/2026.07.13.738177; this version posted July 14, 2026. The copyright holder for this preprint (which was not certified by peer review) is the author/funder, who has granted bioRxiv a license to display the preprint in perpetuity. It is made available under a CC-BY 4.0 International license.

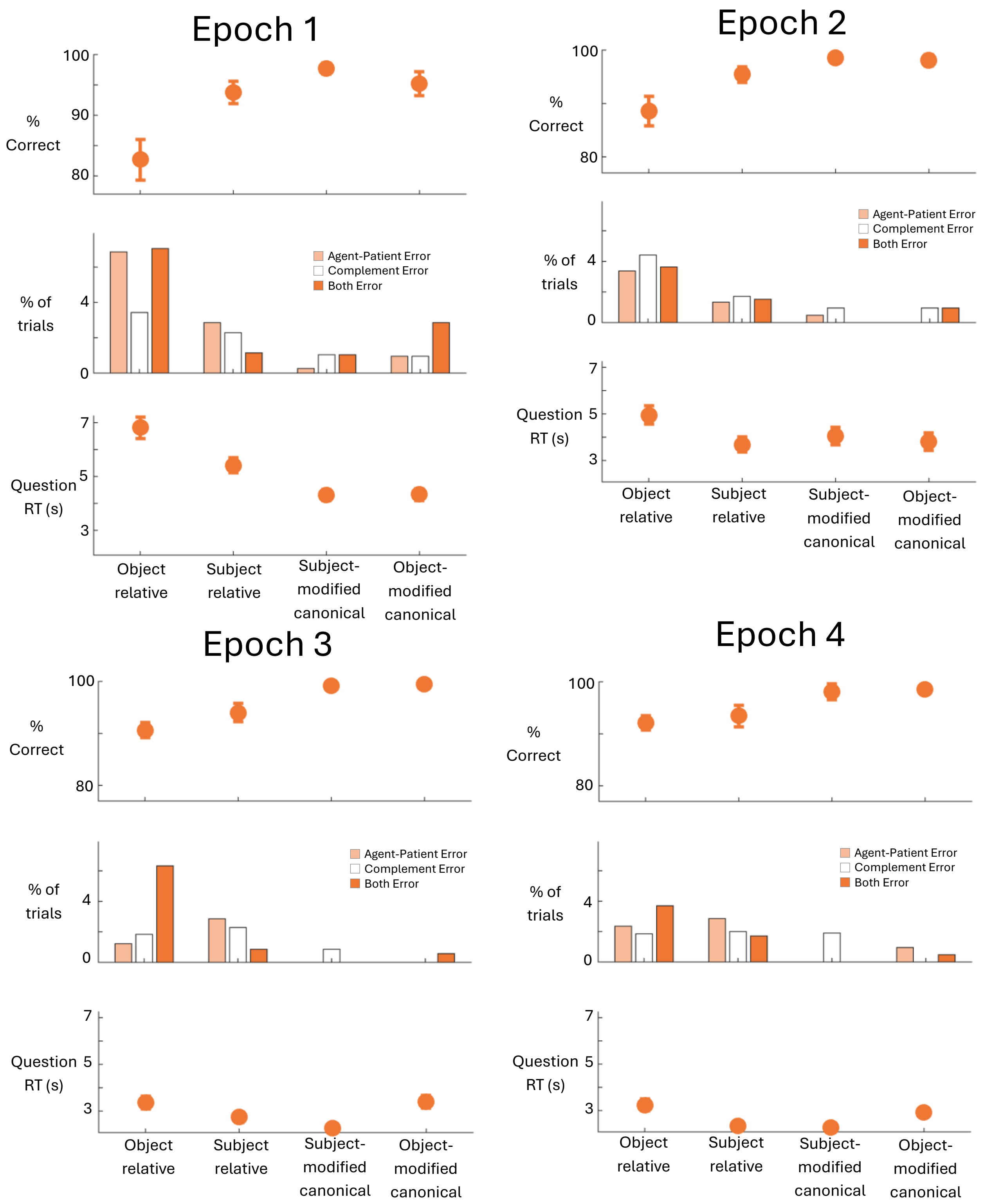

Figure S2. The graphs compare four different succeeding epochs across the four sentence structures: object relative, subject relative, subject-modified canonical, and object-modified canonical. Each epoch contains three graphs comparing behavior response to the comprehension questions. The topmost graph describes the percentage of accuracy for the comprehension questions. The middle graph describes the percent of trials that exhibited a agent-patient error or complement error, or both. The bottommost graph describes the response time of answering the comprehension questions.

bioRxiv preprint doi: https://doi.org/10.64898/2026.07.13.738177; this version posted July 14, 2026. The copyright holder for this preprint (which was not certified by peer review) is the author/funder, who has granted bioRxiv a license to display the preprint in perpetuity. It is made available under a CC-BY 4.0 International license.

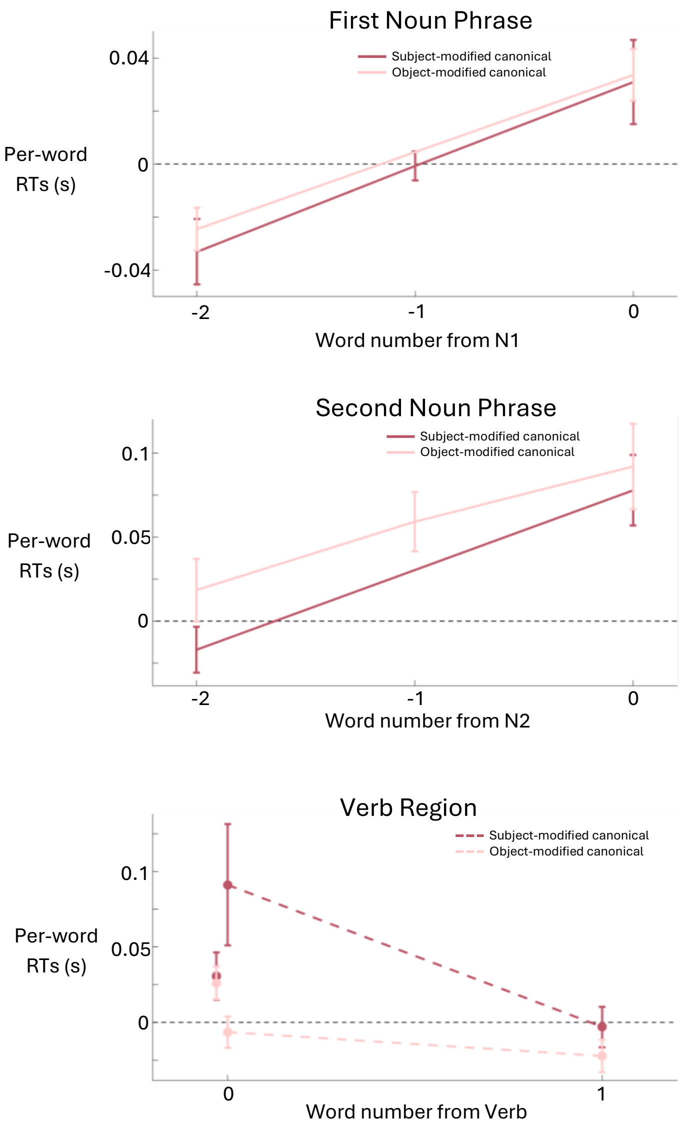

Figure S3. The three graphs describe the baseline-subtracted raw (not residualized) per-word response times for subject-modified canonical and object-modified canonical sentences. The top-left graph describes the RTs for the word number from the first noun phrase (N1). The bottom-left graph describes the RTs from the second noun phrase (N2). The top-right graph describes the word number from the verb.

bioRxiv preprint doi: https://doi.org/10.64898/2026.07.13.738177; this version posted July 14, 2026. The copyright holder for this preprint (which was not certified by peer review) is the author/funder, who has granted bioRxiv a license to display the preprint in perpetuity. It is made available under a CC-BY 4.0 International license.

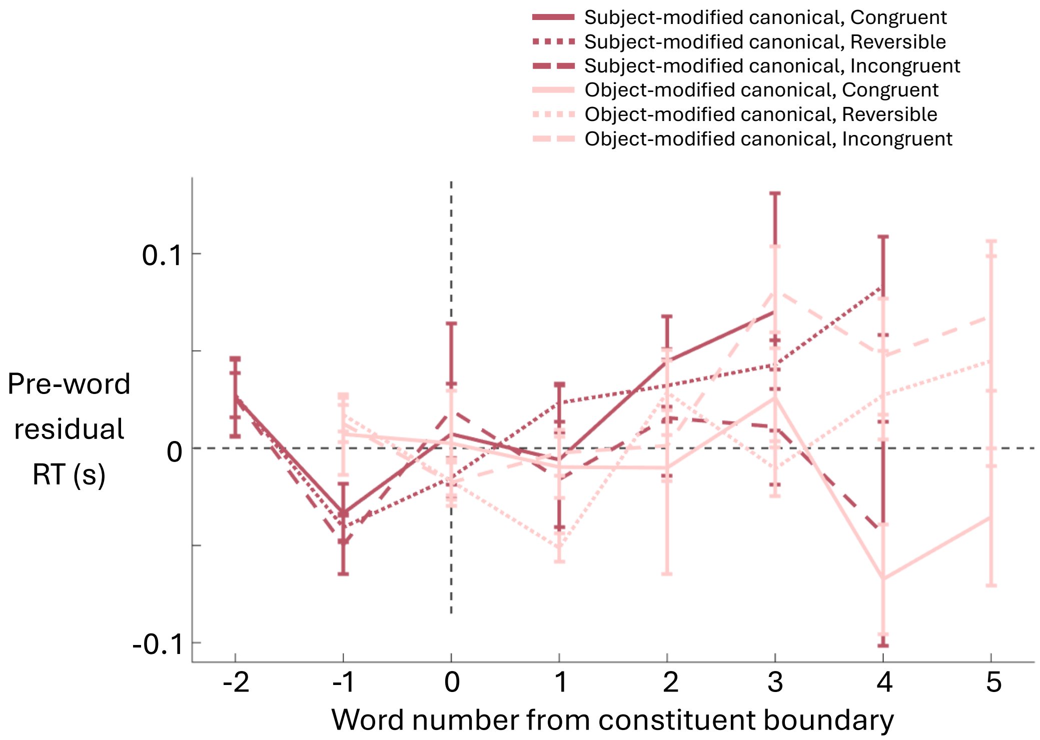

Figure S4. The graph describes the per-word residual response times by the word number distance from the constituent boundary for the subject-modified canonical and object-modified canonical sentence structures and their plausibility (congruent, reversible, incongruent).
